# Uncommon Extrapyramidal Reaction to Ondansetron

**DOI:** 10.7759/cureus.80362

**Published:** 2025-03-10

**Authors:** Carolina S Dias, Catarina Moreira, João Gaspar, Maria Santos

**Affiliations:** 1 Anesthesiology and Critical Care, Instituto Português de Oncologia do Porto Francisco Gentil, Porto, PRT; 2 Medical Oncology, Instituto Português de Oncologia do Porto Francisco Gentil, Porto, PRT

**Keywords:** adverse side effect, antiemetics, extrapyramidal syndrome, movement disorder, prescription drugs

## Abstract

We describe the case of a 77-year-old man, who was admitted for the initiation of chemotherapy for stage IV small cell lung cancer without cerebral metastases. On the first day post-admission, the patient developed altered mental status and right-sided weakness after premedication with ondansetron and dexamethasone. Symptoms resolved spontaneously within 90 minutes. An ischemic event was suspected despite unremarkable brain imaging. Prophylactic anticoagulation and statins were initiated.

Five days later, a second episode of altered mental status, anisocoria, facial paresis, and severe dysarthria occurred following ondansetron administration. Bloodwork revealed significant hyponatremia (116 mEq/L), suggesting Syndrome of Inappropriate Antidiuretic Hormone Secretion (SIADH), despite this suspicion, there was no further investigation to confirm this diagnosis due to rapid symptom reversal after biperiden administration, implicating an adverse reaction to ondansetron.

Ondansetron and metoclopramide were discontinued, hyponatremia was corrected, and no further episodes occurred during the hospital stay. This case highlights the necessity of awareness for adverse reactions of ondansetron that can mimic cerebrovascular events and the need for a thorough revision of medication in complex cases.

## Introduction

Ondansetron is a widely used 5-HT3 receptor antagonist, commonly employed in the treatment and prevention of nausea and vomiting during the perioperative period and across various chemotherapy regimens [1-3]. The most frequently reported neurological adverse effects include fatigue, headache, and malaise [2].

There is no consensus on the mechanism responsible for these symptoms, although some studies suggest a correlation with repetitive administration and dosing. Additionally, research indicates that ondansetron may inhibit or reduce increased mesolimbic dopamine activity and counteract the heightened locomotor activity caused by mesolimbic dopamine excess [4].

Extrapyramidal reactions to antiemetic drugs have been reported, though they are most commonly associated with metoclopramide administration. Such reactions following ondansetron administration are extremely rare, with the existing literature limited to a few case reports published several years ago, and no recent updates on this topic. The most common treatment options described in literature for this episode are diphenhydramine and biperiden [4,5]. The available reports primarily describe dystonic reactions, some of which are associated with tonic-clonic movements, mainly occurring after emergence from general anesthesia [5,6]. The most frequent extrapyramidal effects include dystonia, oculogyric crises and involuntary movements of muscles in extremities [5,7]. There is no report on the incidence of extrapyramidal reactions to ondansetron but an incidence of 0,2% is estimated to metoclopramide [5]. The most recent case reported in the literature describes the onset of abnormal posturing and involuntary repetitive tonic movements following emergence from anesthesia [7].

Although these reactions may present with mild symptoms, some cases can be life-threatening, particularly when airway patency is compromised or when movement disorders result in injuries. Given these risks, it is crucial to rule out other significant neurological disorders and maintain a high index of suspicion to ensure prompt diagnosis and treatment.

## Case presentation

A 77-year-old man was admitted to the oncology ward to receive the first cycle of chemotherapy for stage IV small cell lung cancer without cerebral metastases. The patient was autonomous, and his medical history was notable for a past smoking habit (now ceased), hypertension, benign prostatic hyperplasia, and hyperuricemia.

On the first day of hospitalization, approximately 30 minutes after the administration of chemotherapy premedication with 4 mg of intravenous ondansetron and 8 mg of intravenous dexamethasone, and before chemotherapy had begun, the medical emergency team was activated due to a sudden change in the patient’s mental status while he was having lunch. The patient, who was accompanied by a family member at the time, progressively became somnolent.

On physical examination, an altered mental status and right-sided weakness were observed, while hemodynamic and respiratory stability were maintained. The patient was responsive only to painful stimuli, localizing pain with both extremities but exhibiting reduced mobility on the right side, accompanied by right facial paresis. The plantar reflexes were bilaterally flexor. Both eyes were positioned at the midline; the left pupil was irregular, possibly due to prior surgery, while the right pupil was miotic and reactive to light.

An electrocardiogram was performed, revealing sinus bradycardia with a first-degree atrioventricular block. Blood gas analysis showed no respiratory, metabolic, or electrolyte abnormalities that could explain the patient's clinical condition.

**Figure 1:**
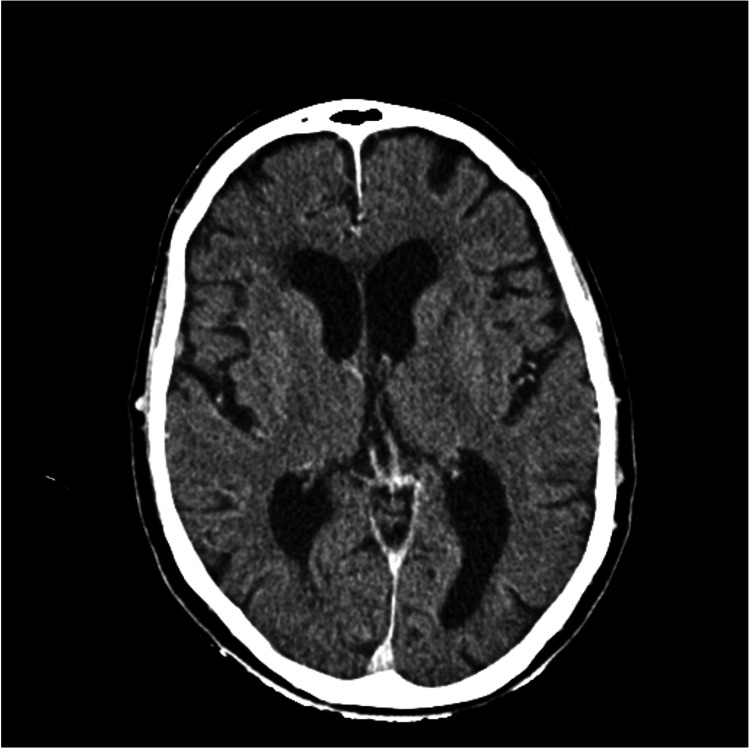
Computed tomography angiography of the cerebral vessels after the first event.

**Figure 2:**
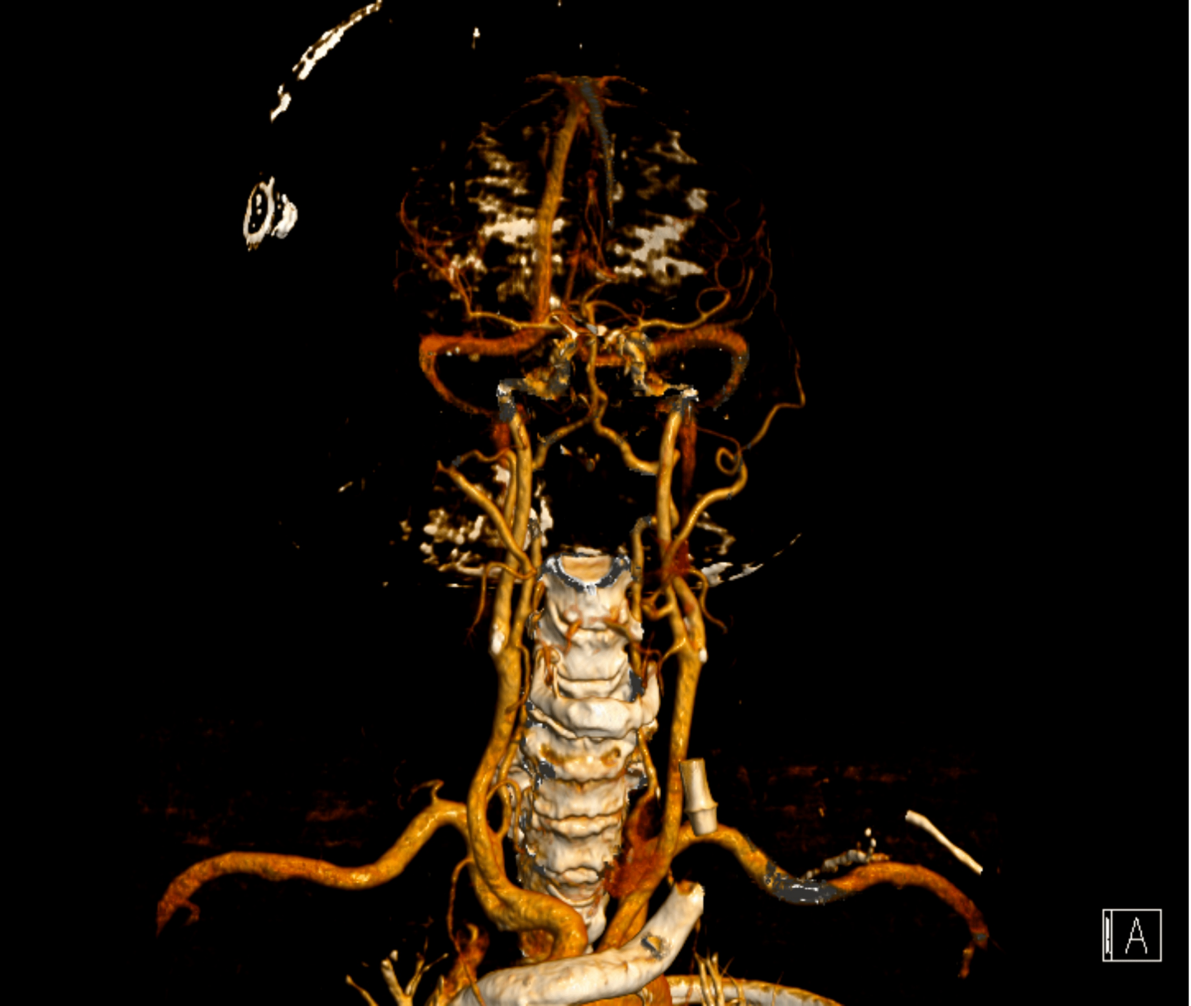
Cerebral vessels reconstruction of computed tomography angiography after the first event.

The patient promptly underwent a brain CT angiography, which did not reveal any vascular lesions or thrombi in major vessels (Figures 1, 2).

Approximately one hour after the onset of symptoms, the patient gradually became more alert, and by one hour and thirty minutes after onset, he had returned to his baseline mental status without any neurological deficits.

**Figure 3:**
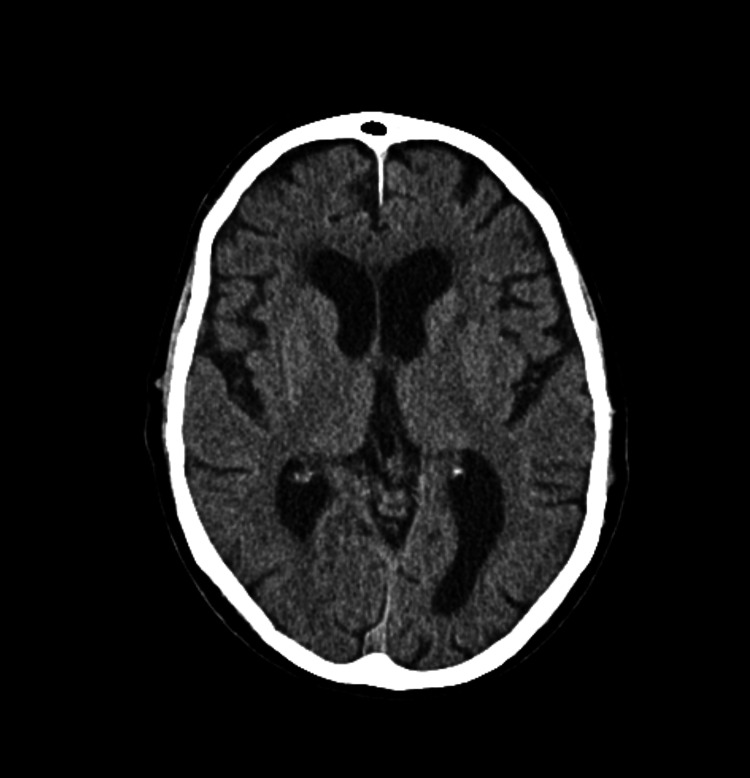
Control computed tomography angiography of the cerebral vessels 24 hours after the first event.

Due to the suspicion of a transient ischemic event as the primary cause of the symptoms, a loading dose of 300 mg of aspirin was administered, followed by a daily dose of 100 mg of aspirin. Prophylactic low-molecular-weight heparin and a statin were also initiated. The following day, a follow-up brain CT was performed to exclude ischemic lesions that may not have been detected in the initial scan, and no evidence of new ischemic areas was found (Figure 3).

The patient was kept in the hospital under close neurological monitoring, and 10 mg of intravenous metoclopramide every eight hours was added to the antiemetic regimen with ondansetron and dexamethasone due to persistent nausea and vomiting.

Five days after the first episode, shortly after the administration of 4 mg intravenous ondansetron for nausea before breakfast, the patient again developed altered mental status, responsive only to pain stimuli, with marked somnolence, anisocoria, central facial paresis, and severe dysarthria. There were no signs of motor deficits in the extremities.

**Figure 4:**
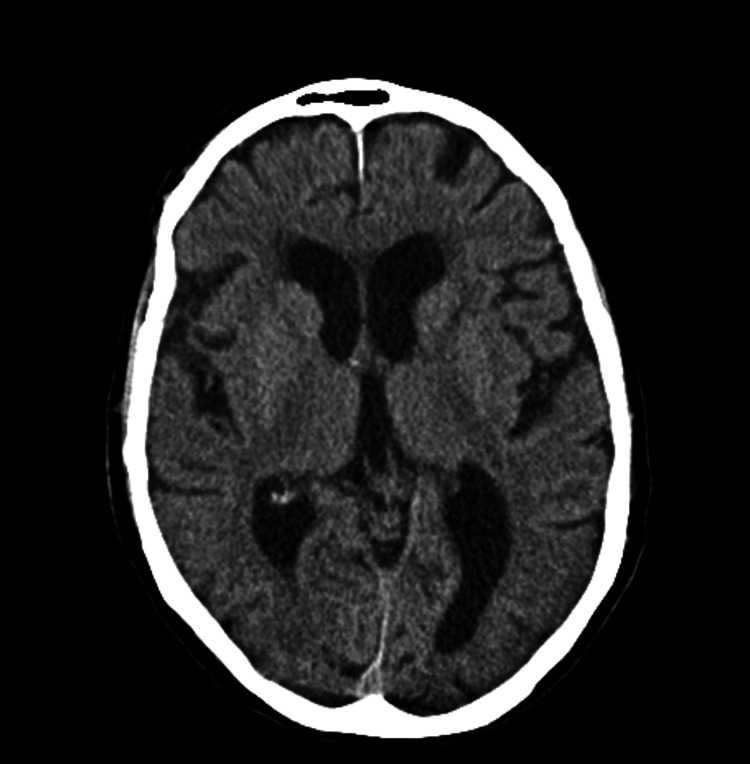
Computed tomography angiography of the cerebral vessels after the second event.

**Figure 5:**
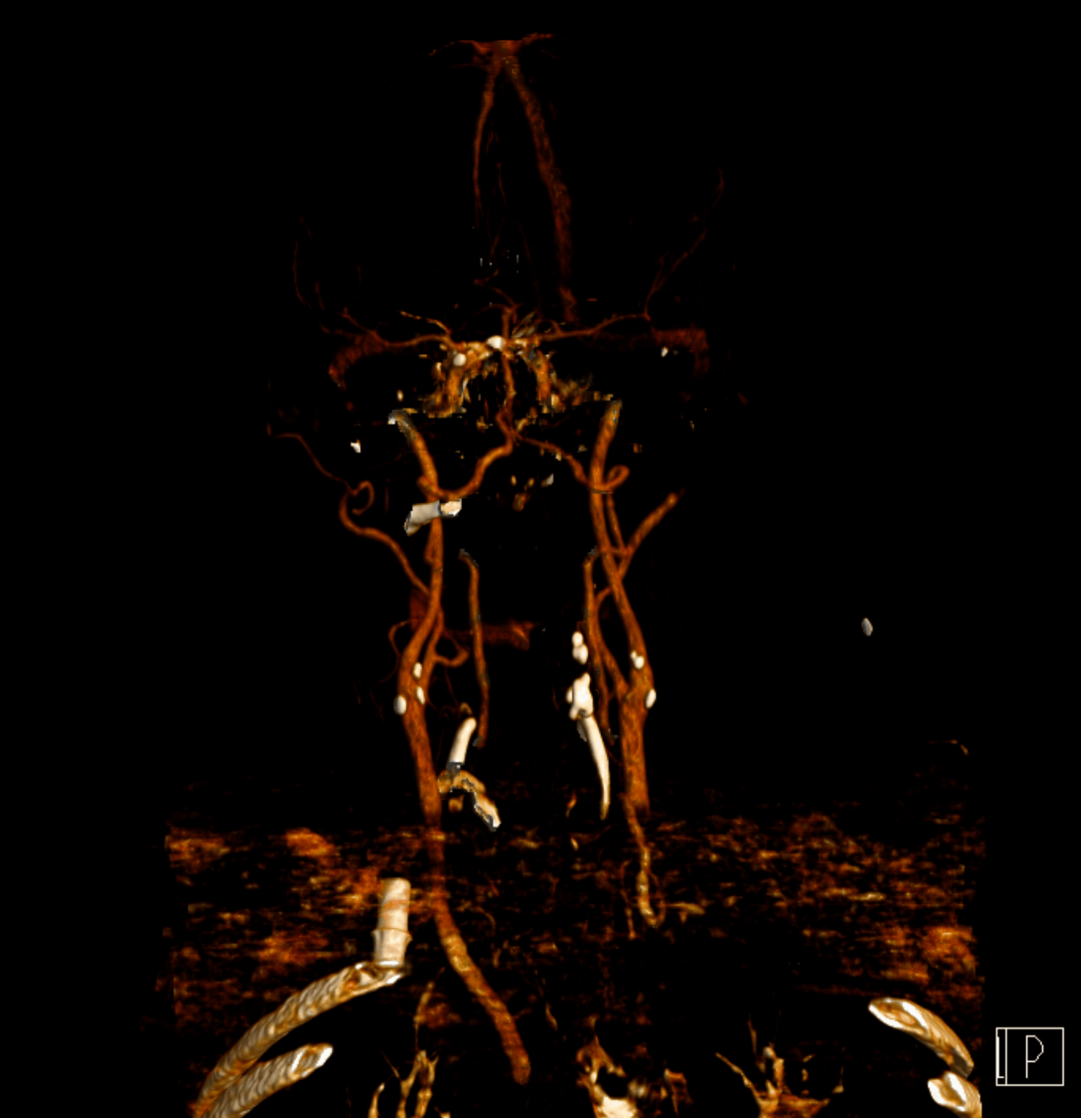
Cerebral vessels reconstruction of computed tomography angiography after the second event.

A transient ischemic event was once again suspected, and a brain CT angiography was performed, which again showed no evidence of acute ischemic lesions or major vessel occlusion (Figures 4, 5). However, on this occasion, the blood gas analysis revealed significant hyponatremia of 116 mEq/L, raising concerns about a possible diagnosis of Syndrome of Inappropriate Antidiuretic Hormone Secretion (SIADH).

A review of the patient's previous medications was conducted, and the nurse emphasized that the only medications administered prior to the episodes were ondansetron and dexamethasone, leading to the primary suspicion of an adverse reaction to ondansetron.

The patient was admitted for observation in the ICU. Given the primary suspicion of an adverse reaction to ondansetron, the first therapeutic measure was the administration of a 2 mg bolus of biperiden, resulting in partial reversal of the episode. This was followed by another 2 mg bolus, leading to rapid resolution of the symptoms and a prompt return to the patient's previous mental status within a few minutes.

Due to the rapid response to treatment, an adverse reaction to ondansetron was assumed, and no further workup to rule out SIADH was conducted. The hyponatremia was corrected overnight in the ICU.

After the reversal of symptoms with biperiden, an adverse reaction to ondansetron was suspected, and the antiemetic prophylaxis was changed. The patient remained overnight in the ICU, where the hyponatremia was corrected, and was discharged to the oncology ward the following day, with ondansetron and metoclopramide discontinued. No further episodes were observed during the remainder of the hospital stay.

**Figure 6:**
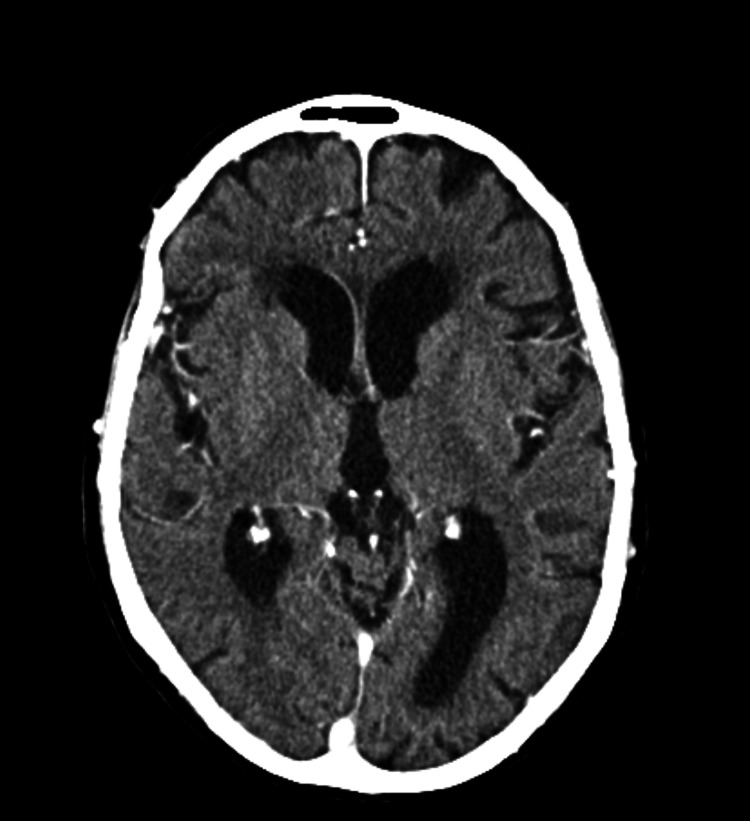
Control computed tomography of the cerebral vessels 30 days after the first event.

Thirty days after the first event, a control CT angiography was made and there was no evidence of ischemic lesions (Figure 6).

## Discussion

Oncologic patients undergoing chemotherapy and radiotherapy have a high incidence of nausea and vomiting. Treatment regimens often include a combination of antiemetics to prevent these side effects. Ondansetron is frequently used for this prophylaxis due to its excellent safety profile and efficacy in the treatment and prevention of chemotherapy-induced emesis [2,8]. Extrapyramidal reactions are more commonly reported following the administration of metoclopramide [1]. The literature on extrapyramidal reactions to ondansetron is limited and mainly consists of a few outdated case reports.

Extrapyramidal reactions to ondansetron can range from mild motor impairment to altered mental status, potentially with airway compromise, posing a life-threatening risk [9,10]. In our case, the symptoms led to altered mental status, prompting the prompt exclusion of an acute, life-threatening cerebral or cardiac event. After ruling out possible reversible causes of the event, other differential diagnoses were considered.

The cases reported in the literature present with varying symptom profiles, including mental status impairment ranging from mild to severe, sometimes with airway compromise requiring ventilatory support, and movement disorders as a common feature [4,9,11]. In our case, the defining characteristic was facial paresis, along with mental status impairment.

In the second episode, there was a confounding factor regarding the aetiology of the symptoms. The patient presented with severe hyponatremia, which could have been indicative of SIADH and might explain the patient's symptoms. While this finding needed to be promptly addressed and treated, a high index of suspicion was essential to establish an adverse reaction to ondansetron as the primary diagnosis. A review of the patient’s prescription and the timing of drug administration was crucial in determining the diagnosis, highlighting the importance of a thorough anamnesis and accurate documentation of medication administration.

The differential diagnosis between extrapyramidal reaction to ondansetron, SIADH, and acute cerebrovascular event requires careful clinical evaluation and specific diagnostic tests. Common signs of SIADH include altered mental status, lethargy, nausea, and vomiting; however, extrapyramidal symptoms such as dystonia, rigidity, and facial paresis are not typically observed. Furthermore, SIADH's onset can be gradual, although it may develop rapidly in some cases. An acute cerebrovascular event typically presents with the sudden onset of focal neurological deficits, such as weakness and dysarthria, with the pattern of neurological deficits often correlating with a specific vascular territory. In our case, the primary suspicion, after normal brain imaging, was an adverse reaction to ondansetron due to the timing of the drug administration and the onset of symptoms.

The incidence of SIADH in small-cell lung cancer is estimated to be approximately 15% according to the literature [12]. In our case, further investigation of the patient’s hyponatremia could have been pursued. However, due to the sudden onset of symptoms and their prompt resolution following biperiden administration, an adverse reaction to ondansetron was assumed as the primary diagnosis. Consequently, the hyponatremia was considered a paraneoplastic manifestation of the lung cancer rather than the underlying cause of the neurological symptoms.

Biperiden is an anticholinergic agent with central effects, commonly used for the treatment of Parkinson’s syndrome and extrapyramidal symptoms resulting from the use of various agents, such as neuroleptics and antipsychotics. There are case reports of biperiden being used to treat extrapyramidal symptoms associated with metoclopramide intake [5,13]. However, our literature review did not identify its use in the context of adverse reactions to ondansetron. Instead, diphenhydramine is typically the drug of choice [4,7].

Oncological patients are often very fragile, with multiple comorbidities, and they undergo aggressive treatments that themselves constitute a significant challenge. Every drug administered to these patients must be carefully considered, weighing the potential risks and benefits.

## Conclusions

Sudden changes in mental status can have multiple aetiologies, and cerebral imaging should be promptly performed. Adverse reactions to ondansetron may mimic acute cerebrovascular events, making it essential to exclude life-threatening conditions through appropriate investigations. In complex patients with confounding factors and multiple potential diagnoses for the same symptoms, a high index of suspicion is crucial. A thorough review of the patient's medication regimen should always be included in the diagnostic process.

Awareness of this rare but possible adverse reaction to ondansetron is crucial to prevent unnecessary diagnostic investigations and inappropriate treatments.

## References

[1] Deegan R (1992). Ondansetron: pharmacology of a specific 5HT3-receptor antagonist. Am J Med Sci.

[2] Piechotta V, Adams A, Haque M (2021). Antiemetics for adults for prevention of nausea and vomiting caused by moderately or highly emetogenic chemotherapy: a network meta-analysis. Cochrane Database Syst Rev.

[3] Roila F, Del Favero A (1995). Ondansetron clinical pharmacokinetics. Clin Pharmacokinet.

[4] Sprung J, Choudhry FM, Hall BA (2003). Extrapyramidal reactions to ondansetron: cross-reactivity between ondansetron and prochlorperazine?. Anesth Analg.

[5] Sheikh Hassan M, Ahmed Nor M (2022). Metoclopramide induced acute dystonic reaction: a case report. Ann Med Surg (Lond).

[6] Mathews HG 3rd, Tancil CG (1996). Extrapyramidal reaction caused by ondansetron. Ann Pharmacother.

[7] Baigent AV, Morris EA (2023). Severe acute drug-induced dystonia in the post-operative period requiring tracheal re-intubation. Anaesth Rep.

[8] Plosker GL, Milne RJ (1992). Ondansetron: a pharmacoeconomic and quality-of-life evaluation of its antiemetic activity in patients receiving cancer chemotherapy. Pharmacoeconomics.

[9] Ritter MJ, Goodman BP, Sprung J, Wijdicks EF (2003). Ondansetron-induced multifocal encephalopathy. Mayo Clin Proc.

[10] Kumar N, Hu WT (2009). Extrapyramidal reaction to ondansetron and propofol. Mov Disord.

[11] Tolan MM, Fuhrman TM, Tsueda K, Lippmann SB (1999). Perioperative extrapyramidal reactions associated with ondansetron. Anesthesiology.

[12] Saeed BO (2009). Syndrome of inappropriate secretion of antidiuretic hormone. South Med J.

[13] Emorinken A, Agbadaola OR (2021). Metoclopramide-induced acute dystonia misdiagnosed as an epileptic seizure in a lupus patient. J Epilepsy Res.

